# Surfactant-free gelatin-stabilised biodegradable polymerised high internal phase emulsions with macroporous structures

**DOI:** 10.3389/fchem.2023.1236944

**Published:** 2023-08-23

**Authors:** Rachel Furmidge, Caitlin E. Jackson, María Fernanda Velázquez de la Paz, Victoria L. Workman, Nicola H. Green, Gwendolen C. Reilly, Vanessa Hearnden, Frederik Claeyssens

**Affiliations:** ^1^ Materials Science and Engineering, The Kroto Research Institute, University of Sheffield, Sheffield, United Kingdom; ^2^ Insigneo Institute for In Silico Medicine, University of Sheffield, Sheffield, United Kingdom

**Keywords:** polyHIPE, gelatin, surfactant-free, polycaprolactone, poly(glycerol sebacate), porous polymers, emulsion templating

## Abstract

High internal phase emulsion (HIPE) templating is a well-established method for the generation of polymeric materials with high porosity (>74%) and degree of interconnectivity. The porosity and pore size can be altered by adjusting parameters during emulsification, which affects the properties of the resulting porous structure. However, there remain challenges for the fabrication of polyHIPEs, including typically small pore sizes (∼20–50 μm) and the use of surfactants, which can limit their use in biological applications. Here, we present the use of gelatin, a natural polymer, during the formation of polyHIPE structures, through the use of two biodegradable polymers, polycaprolactone-methacrylate (PCL-M) and polyglycerol sebacate-methacrylate (PGS-M). When gelatin is used as the internal phase, it is capable of stabilising emulsions without the need for an additional surfactant. Furthermore, by changing the concentration of gelatin within the internal phase, the pore size of the resulting polyHIPE can be tuned. 5% gelatin solution resulted in the largest mean pore size, increasing from 53 μm to 80 μm and 28 μm to 94 µm for PCL-M and PGS-M respectively. In addition, the inclusion of gelatin further increased the mechanical properties of the polyHIPEs and increased the period an emulsion could be stored before polymerisation. Our results demonstrate the potential to use gelatin for the fabrication of surfactant-free polyHIPEs with macroporous structures, with potential applications in tissue engineering, environmental and agricultural industries.

## 1 Introduction

Various polymer applications benefit from having highly porous structures with a high degree of openness and interconnectivity. For example, in tissue engineering this enables cell ingrowth; in filters, interconnectivity facilitates mass transport and for electrode substrates and catalysts high surface areas result in a better current and substrate conversion, respectively (Chong et al., 2019; Elango et al., 2021; Fager et al., 2021; Vásquez et al., 2021; Maksoud et al., 2022; Mravljak et al., 2022). There are numerous methods of introducing porous geometries within polymers, including bioprinting, particulate leaching, freeze drying, electrospinning and emulsion templating (Eltom et al., 2019; Sharma et al., 2022). Emulsion templating is a relatively simple technique that can be easily tuned to control and influence the resulting structures. Additionally, the internal phase can easily be removed from the polymerised structure via washing and/or dissolving, unlike techniques such as salt/particulate leaching where there is a risk that the presence of residual particles may negatively affect biocompatibility (Maksoud et al., 2022; Montanheiro et al., 2022).

Emulsion templating is performed via the mixing of two immiscible fluids, commonly in the presence of a surfactant, to form a water-in-oil (w/o) or oil-in-water (o/w) emulsion (Foudazi, 2021). During mixing droplets of the internal phase are dispersed within the external phase. The external phase is then solidified, with the internal phase droplets acting as templates for the formation of pores. The internal phase is then removed, resulting in a porous structure. Emulsion templating techniques can be easily tuned by altering parameters such as temperature, surfactant type and concentration, stirring speed and the volume fraction of the internal phase (Aldemir Dikici and Claeyssens, 2020). Emulsions with an internal phase volume >74% are classified as high internal phase emulsions (HIPEs) (Mert and Mert, 2022). Following the polymerisation of these HIPEs (polyHIPEs), a porous structure is fabricated with a high degree of porosity and interconnectivity (Aldemir Dikici and Claeyssens, 2020). Interconnectivity in polyHIPEs occurs when the external phase ruptures at the thinnest sections between the densely packed droplets of the internal phase. This results in the formation of “windows,” providing interconnections between pores (Menner and Bismarck, 2006; Silverstein, 2014).

The optimal pore size of a polyHIPE varies greatly depending on the specific application. For example, within tissue engineering, the optimal pore size for angiogenesis has been reported as 160–270 µm (Artel et al., 2011), whereas pore sizes of 11 µm have been reported as optimal for the infiltration of dermal fibroblasts into elastin scaffolds (Rnjak-Kovacina et al., 2011). In membrane filtration systems for conventional particle filtration for water purification, there are a range of optimal pore sizes (5–1,000 µm) for the capture of different particle types (e.g., > 25 µm for sand, 10–100 µm for pollen and <50 µm for atmospheric dust) (Lee et al., 2016). For other filtration applications, such as oil recovery, optimal pore sizes between 82.3 and 145.6 µm have been reported (Zhang and Guo, 2017; Sherborne and Claeyssens, 2021). Despite the advantages of HIPE templating, the pore size of surfactant-stabilised polyHIPEs is typically quite small, <50 μm (Barbetta and Cameron, 2004; Dikici et al., 2019), with smaller windows (1–10 μm) forming interconnections between pores (Silverstein, 2014; Sun et al., 2019). Given the ranges of pore sizes required for these different applications, it is beneficial to be able to alter the pore size of polyHIPE materials.

A known mechanism for increasing the pore size of emulsion-templated materials is via the modulation of emulsion stability (Dhavalikar et al., 2021). Emulsions are thermodynamically unstable, and as such, it is energetically favourable for the surface area of the internal droplets to be reduced if the interfacial tension is too high. This mechanism occurs through the coalescence of droplets, increasing droplet size to reduce surface area, thus resulting in increased pore size of the polymerised emulsion (polyHIPE) (Bokhari et al., 2007; Ravera et al., 2021). Through this principle, reducing emulsion stability can be used to increase the pore size of emulsion-templated materials. Previous attempts have been made to reduce the stability of emulsions, for example, Kent and Saunders reported the use of magnesium sulphate within w/o emulsions to reduce the adsorption of surfactant, thus increasing the droplet size within the emulsion (Kent and Saunders, 2001; Aldemir Dikici and Claeyssens, 2020). Concerning emulsion-templated biomaterials, Choi et al. (2010) created porous poly (D, L-lactide-co-glycolide) (PLGA) beads using gelatin, with tuneable pore size, controlled through the use of phase-separated emulsions. To increase pore size within the beads, fractions of the emulsion with reduced stability were used during bead fabrication (Choi et al., 2010).

As previously mentioned, surfactant type and concentration are also important parameters in the fabrication of emulsions and play a key role in the resulting pore size of the polyHIPE. Surfactants used to create polyHIPEs are commonly amphiphilic, consisting of a hydrophilic head group and a hydrophobic tail. The surfactant forms a barrier between the droplet and the surrounding phase, reducing the surface tension and facilitating the interaction between the two phases. However, due to the synthetic nature of most commonly used surfactants, their use can lead to cytotoxicity, and/or a series of lengthy and costly washing steps. The use of surfactant-free templating methods, such as Pickering HIPEs (e.g., soft and Janus particles) (Venkataramani et al., 2020) as well as biologically-based surfactants (Wang et al., 2020; Chen et al., 2021) have been previously explored. Biologically-based surfactants of plant and microbial origin have been explored by the agricultural, chemical, and cosmetics industries (Duprat-De-Paule et al., 2018; Deotale et al., 2019; Moldes et al., 2021; Gayathiri et al., 2022). However, one of their main challenges is high production costs (Farias et al., 2021).

Gelatin is a natural polymer formed by the denaturation of collagen via partial hydrolysis (Zhang et al., 2020). Gelatin undergoes a sol-gel transition when dissolved in water at temperatures between 35°C and 37°C, wherein cooling of the gelatin solution induces the formation of triple helices stabilised by intermolecular hydrogen bonds (Haug et al., 2009), allowing the reversible formation of gels (Chen and Vyazovkin, 2009). Gelatins have reportedly been used as an emulsifier for many different applications, commonly in the food industry, where they serve as foaming, emulsifying and wetting agents to improve the quality of various foods and improve their stabilisation (Karim and Bhat, 2009; Zhang et al., 2020). Gelatin exhibits amphiphilic behaviour and can decrease interfacial tension by migrating from the water phase to the oil/water interface (Ding et al., 2020). However, gelatin is viewed as a weak stabiliser, especially when compared to other surfactants commonly used to make polyHIPEs, such as Span80, Hypermer B246 and polyglycerol polyricinoleate (PGPR) (Aldemir Dikici and Claeyssens, 2020; Zhang et al., 2020). Due to the emulsification properties of gelatin, we postulated that gelatin could be used to fabricate surfactant-free polyHIPEs, and, as the emulsifying ability of gelatin is weak, these polyHIPEs would have large pore sizes (>50 µm).

This study utilises gelatin solutions as the internal phase in polymer-based emulsions to fabricate surfactant-free polyHIPEs. We assessed the pore geometry and characteristics of the resulting polyHIPEs compared to conventional polyHIPEs fabricated with water as the internal phase. The concentration of gelatin was varied to assess the effect on polyHIPE structure. In addition, we investigated the effect of using gelatin in combination with a commonly used surfactant (Hypermer B246) to further elucidate the behaviour of gelatin as a stabiliser.

For these experiments, we have used two synthetic biodegradable polymers, polycaprolactone-methacrylate (PCL-M) and poly(glycerol sebacate)-methacrylate (PGS-M). PCL is FDA-approved and the photocurable form, PCL-M, has been extensively researched within our group for bone and nerve tissue engineering applications (Aldemir Dikici et al., 2020; Field et al., 2021; Aldemir Dikici et al., 2022a). PGS is an emergent material that has been well-documented as being softer than most traditionally used synthetic polymers (Pashneh-Tala et al., 2018; Vogt et al., 2021). PCL-M and PGS-M were selected as both materials are biodegradable and biocompatible, whilst having different chemical and mechanical profiles (Labet and Thielemans, 2009; Rai et al., 2012).

The long-term stability of the fabricated emulsions was assessed over the course of 2 months, and the resulting polyHIPEs were characterised using scanning electron microscopy (SEM). Finally, the effect of using gelatin during polyHIPE fabrication on the mechanical properties of the polyHIPEs was investigated. Overall, this investigation highlights the potential use of gelatin as a surfactant-free method to generate polyHIPEs with large pore sizes in polymeric constructs.

## 2 Materials

Photoinitiator (2,4,6-Trimethylbenzoyl Phosphine Oxide/2-Hydroxy-2- Methylpropiophenone blend), glycerol (99%), sebacic acid (99%), 4-methoxyphenol (99%), trimethylamine (99.5%), methacrylic anhydride (94%, MEA) and type A gelatin from porcine skin (300 g Bloom) were purchased from Sigma Aldrich. Chloroform (99%), toluene (99.5%), ethanol (99%), dichloromethane (99%, DCM), hydrochloric acid (37%) and glacial acetic acid (99%) were purchased from Fisher Scientific. The surfactant, Hypermer B246 (98%) was received as a sample from Croda (Goole, United Kingdom). High molecular weight 4-arm methacrylated polycaprolactone [PCL-M, 95% degree of methacrylation, M_w_ = 20,331 g/mol, Supplementary Figures S1C, E) was synthesised in the laboratory [a general synthesis method is given in Aldemir Dikici et al. (2019)].

## 3 Methods

### 3.1 PGS-M synthesis

PGS pre-polymer was synthesised via methods previously described (Pashneh-Tala et al., 2018; Singh et al., 2018; Becerril-Rodriguez and Claeyssens, 2022). Briefly, glycerol and sebacic acid in an equimolar ratio were stirred and heated to 120°C with nitrogen gas at a flow rate of 1 L/min applied to the system for 24 h to prevent oxygen contamination. After 24 h, a vacuum, at a pressure of 9 mbar, was applied for a further 24 h to remove excess water from the system following the polycondensation reaction.

To methacrylate PGS to enable photocuring, PGS prepolymer was dissolved in DCM 1:4 (w/v) and 1 mg of the accelerator 4-methoxyphenol was added per gram of PGS-prepolymer to increase the initial rate and extent of polymerisation. The system was then surrounded by an ice bath (0°C) to slow reaction kinetics, and triethylamine was added at a concentration of 0.8 mol/mol of PGS pre-polymer OH groups (for a theoretical 80% degree of methacrylation), followed by an equimolar amount of MEA which was added dropwise. Following the addition of MEA, the temperature was allowed to rise to room temperature over the following 24 h. The actual degree of methacrylation following characterization was 75%, and the molecular weight was 2,065 g/mol (Supplementary Figures S1D, E).

After 24 h, an additional 1 mg 4-methoxyphenol per gram of PGS prepolymer was added to stop the reaction. To remove any residual reagents used during methacrylation, the yielded PGS-M pre-polymer was washed with 30 mM hydrochloric acid. Vacuum filtration through a 6 μm pore cellulose filter (Whatman—Grade 3, GE Healthcare Life Sciences, United Kingdom) was used to remove any potential residual solids before the residual solvent was removed via rotary evaporation for 3 h under a vacuum at a pressure of 9 mbar at approximately 10°C. The residual PGS-M pre-polymer was then removed and stored at −20°C before use.

### 3.2 PCL-M polyHIPE fabrication

0.4 g PCL-M and 0.04 g surfactant were heated to melt the surfactant and PCL-M. 0.6 g of a 60 wt% chloroform and 40 wt% toluene solvent mixture (0.24 and 0.28 mL respectively) and 0.03 g photoinitiator were added to the PCL-M-surfactant mixture respectively. The contents were mixed (250 rpm) using a magnetic stirrer (20 mm × 7 mm) for 3 min at 37°C. Once homogeneous, 2 mL of internal phase (deionized water or gelatin solution) was added dropwise at a rate of approximately 1 droplet/s and the emulsion was further mixed for 5 min.

### 3.3 PGS-M polyHIPE fabrication

0.5 g PGS-M pre-polymer with 0.05 g surfactant was heated to melt the surfactant and reduce polymer viscosity. 0.5 g toluene was then added and then mixed for a minimum of 3 min at 37°C at 250 rpm using a magnetic stirrer (20 mm × 7 mm). Once homogenous, 4 mL of the internal phase (deionised water or gelatin solution) was added into the stirring mixture dropwise at a rate of approximately 1 droplet/s. The mixture was stirred for 3 min to allow the emulsion to thicken before adding 0.05 g of photoinitiator and stirring for a further 2 min.

The internal phase, heated to 37°C before addition to the emulsion, consisted of either deionised water, or a solution of gelatin (5, 7, 10 wt/v%), and emulsions were made either with or without 10 wt/wt% surfactant. The composition of the various emulsions tested can be seen in Table 1.

**TABLE 1:** Emulsion Formulations. Emulsions were fabricated either with or without surfactant, with water or gelatin of varying concentrations as the internal phase.

Emulsion	Gelatin solution concentration (%)	Surfactant (weight (%) of polymer weight)
G0S10	0	10
G5S10	5	10
G7S10	7	10
G10S10	10	10
G0S0	0	0
G5S0	5	0
G7S0	7	0
G10S0	10	0

### 3.4 Polymerisation of PCL-M and PGS-M HIPEs

Emulsions were polymerised via radical polymerisation (Supplementary Figure S2) in a transparent 2 mL syringe. All samples were cured using ultraviolet (UV) light for 5 min on both sides using the OmniCure Series 1,000 system (100 W, Lumen Dynamics, Canada), with 18 W/cm^2^ reported light density and spectral output from 250 to 600 nm. The resulting polyHIPEs were removed from the syringe and washed in 100% ethanol for 2 days, changing the ethanol after a 24 h period. Following this, ethanol was replaced daily for 3 days, rehydrating the polyHIPEs through a series of ethanol dilutions; 70%, 50%, and 25% before placing the polyHIPEs into deionised water. PolyHIPEs were rehydrated gradually in order to prevent severe structural changes due to changes in surface tension during washing. All polyHIPE samples were washed and stored at room temperature. PolyHIPE length and diameter were measured post-curing and after washing to determine the shrinkage of the constructs following the washing process.

To analyse the effect of gelatin on the pore structure of the polymer alone, rather than in the presence of gelatin, before scanning electron microscopy (SEM), helium pycnometry and mechanical testing, polyHIPEs (including those fabricated without gelatin) were washed twice in 99% glacial acetic acid for 15 min at 37°C to remove gelatin before the washing steps outlined above. Following completion of washing, samples were freeze-dried (Lyotrap, LTE Scientific Ltd., United Kingdom) for 24 h to dehydrate the samples before characterisation.

### 3.5 Assessment of polyHIPE pore structure via SEM

To observe and analyse the microstructure of the polyHIPE samples, constructs polymerised within the 2 mL syringe were manually sliced into approximately 1 mm thick discs using a scalpel. The morphology of the polyHIPEs was analysed using a scanning electron microscope (SEM, Inspect F, FEI, United States). Samples were subject to the deposition of gold coating. To avoid surface charging and damage to the sample a low accelerating voltage of 5 kV with a spot size of 3 and a typical vacuum pressure of 10^−5^ mbar at a working distance of 10 mm was applied. The SEM images were used to calculate the average pore size and distribution. Across three micrographs, 60 pores were randomly selected (20 per image) and measured using ImageJ v. 1.48 from the National Institutes of Health (NIH, Bethesda, MD, United States). The pores were selected by placing a 30-square grid over the image and measuring the diameter of each pore that was in contact with each cross-section of the grid. A correction factor (Dhavalikar et al., 2021) was applied to adjust for the assumption that each pore had not been exactly bisected (Barbetta and Cameron, 2004).

### 3.6 Helium pycnometry

The porosity of the PCL-M and PGS-M polyHIPEs produced using different emulsion formulations was determined using a helium pycnometer (AccuPyc 1,340, Micromeritics, United States). In this study, porosity refers to the percentage of void space in the material. Constructs were prepared and freeze-dried as described previously in Section 3.4. The length and diameter of the dry constructs were then measured using digital callipers, which were used to calculate the bulk volume of the polyHIPEs, without factoring in the internal porosity. The polyHIPEs were then placed within a 1 cm^3^ chamber insert within the pycnometer before the chamber was pressurised at 19,500 psi with helium, and the volume of the chamber occupied by the polyHIPE was measured, factoring in the internal porosity. This was the true volume of the constructs, including the porosity, denoted as the “pycnometric volume”. The following equation (Eq. 1) was used to determine the construct porosity:

$$Porosity\;\left(\%\right)=\frac{V_{b}-V_{p}}{V_{b}}*100$$ (1)

where V_b_ was the bulk volume, and V_p_ was the pycnometric volume. Theoretical porosity was calculated using the following formula (Eq. 2):

$$Porosity\;\left(\%\right)=\frac{V_{i}}{V_{i}+V_{e}}$$ (2)

where V_i_ and V_e_ represent the volume of the internal phase (water or gelatin solution) and external phase (PGS-M pre-polymer, surfactant and photoinitiator) respectively. Solvent volume was excluded from internal phase volume as it is presumed that solvent would evaporate following polymerisation of the HIPEs, and thus would not form part of the solid volume.

### 3.7 Long-term stability

G0S10, G5S10, and G5S0 emulsions were fabricated (as described previously, Section 3.2 and Section 3.3) and sealed in an air-tight vial (to maintain humidity), covered in aluminium foil and stored at room temperature. Emulsions were then polymerised using UV light (as described previously, Section 3.4) on day 1, day 7, day 14, and day 56. Following polymerisation all the samples were washed in 100% ethanol for 2 days, changing the ethanol after a 24 h period. Following this, ethanol was replaced daily, rehydrating the polyHIPEs through a series of ethanol dilutions; 70%, 50%, and 25% before placing the polyHIPEs into deionised water. All polyHIPE samples were washed and stored at room temperature. To prepare the samples for SEM analysis, the samples were further washed twice in 99% glacial acetic acid for 15 min at 37°C to remove gelatin before repeating the ethanol and water washing procedure as outlined above.

### 3.8 Mechanical characterisation

The compressive modulus of the PCL-M and PGS-M polyHIPEs was calculated using compressive mechanical testing (MultiTest 2.5–dV, Mecmesin, Slinford, United Kingdom), using the 250 N load cell at room temperature and 40% humidity. Samples were cut into cylinders approximately 1 cm in length before their exact length and diameters were measured using digital callipers for calculating mechanical properties. The polyHIPEs were then placed between two compression plates, and compressive tests were performed on each sample at a rate of 1 N/s until the maximum load of 250 N was reached. The stiffness was calculated from the gradient of the initial linear region of the stress-strain curve for each sample.

### 3.9 Statistical analysis

Statistical analysis was carried out using statistical analysis software (GraphPad Prism, Version 9.4.1, CA, United States). A normality test was used to determine if data was normally distributed. Normally distributed data were analysed using a one-way Brown-Forsythe and Welch analysis of variance (ANOVA) followed by Dunnet’s T3 multiple comparison test. Non-normally distributed data were analysed using a Kruskal–Wallis test with Dunn’s multiple comparisons test. Error bars on graphs indicate standard deviation and the number of technical repeats (*n*) are given in figure captions where applicable. Statistical significance on graphs is represented as *p*-value <0.033 (*), 0.002 (**), and 0.001 (***).

## 4 Results

### 4.1 Manufacturing and assessment of polyHIPE pore structure

HIPEs made with 3%–15% gelatin solution with or without surfactant were assessed. Formulations with <5% gelatin solution without surfactant were not able to form emulsions. In addition, without the presence of gelatin or surfactant, emulsions could not form, thus further assessment of these HIPEs was excluded. Gelatin solutions ≥15% resulted in highly viscous solutions that rapidly gelled, preventing the fabrication of reproducible emulsions. Therefore, stable HIPEs were fabricated using 5, 7%, and 10% gelatin solutions as the internal phase, with and without the addition of surfactant. HIPEs fabricated with gelatin solution as the internal phase appeared to have increased viscosity compared to surfactant-only HIPEs.

**FIGURE 1:**
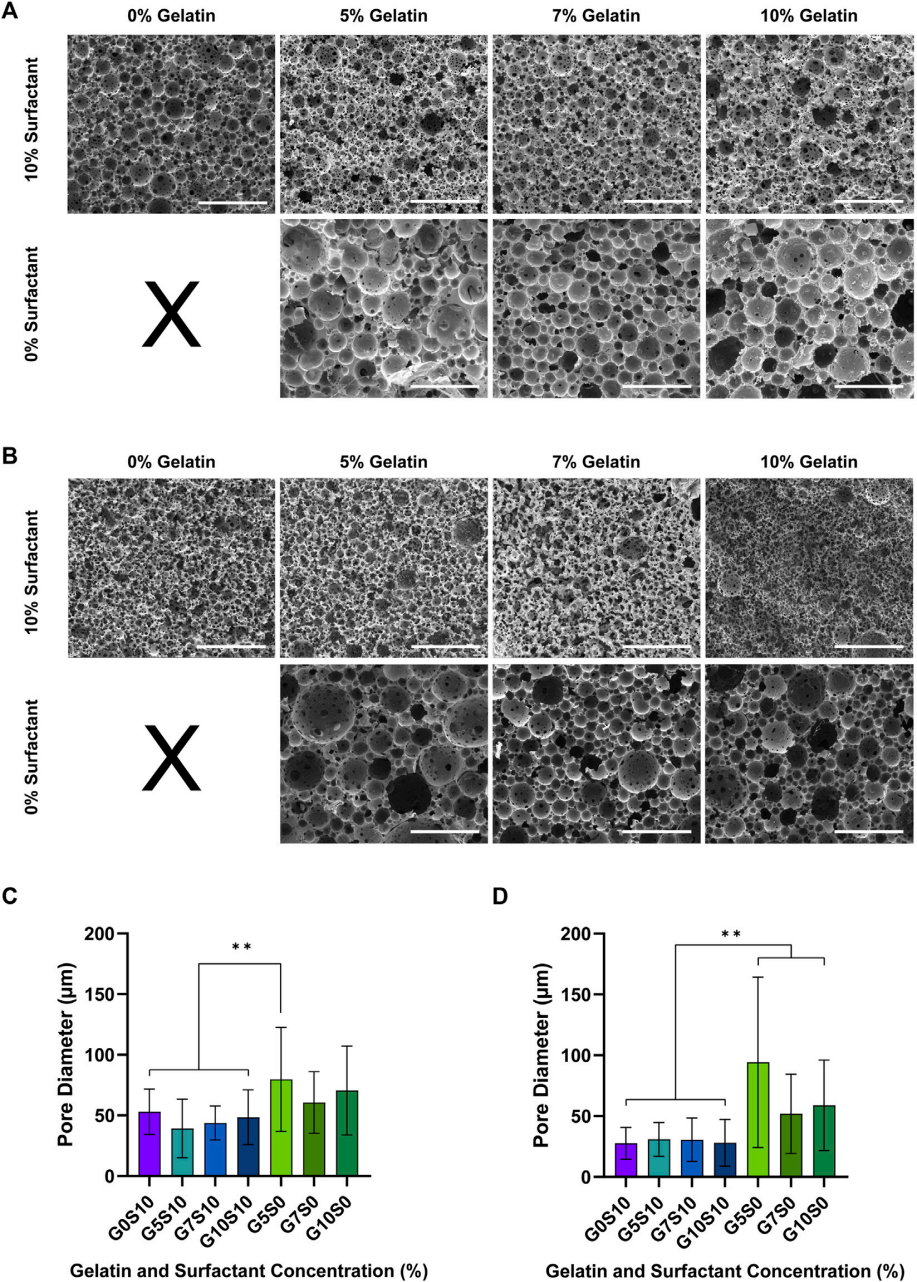
PCL-M and PGS-M polyHIPEs were fabricated using different concentrations of gelatin with or without surfactant. Scanning electron micrographs of **(A)** PCL-M and **(B)** PGS-M polyHIPEs fabricated using 0, 5, 7%, and 10% gelatin solution with or without 10% surfactant (scale bar = 200 µm). Varying the concentration of gelatin within the internal phase of the emulsions affects the resulting pore size of **(C)** PCL-M and **(D)** PGS-M polyHIPEs (mean ± SD, *n =* 60, **p < 0.002). Histograms showing relative frequency and distribution of pore sizes can be found in Supplementary Figures S3, S4 for PCL-M and PGS-M respectively.

Overall, the inclusion of gelatin in the internal phase of the HIPE without the use of additional surfactant increased the pore size of PCL-M and PGS-M polyHIPEs compared to those fabricated with surfactant. A visual change in polyHIPE structure was observed, where there was an increased number of large pores, occupying a greater proportion of the field of view (Figures 1A, B).

Increasing the concentration of gelatin in surfactant-free polyHIPEs resulted in a large distribution of pore sizes (Supplementary Figures S3, S4) which did not yield a statistically significant change in the mean pore size. However, there were trends in the data that correlate to the visual observations from the SEM images. For PCL-M, increasing the concentration of the gelatin solution in surfactant-free polyHIPEs from 5% to 7% resulted in a decrease in mean pore size (79.9 ± 42.9 µm to 60.6 ± 25.4 µm respectively) (Figure 1C). However, further increasing the gelatin concentration from 7% to 10% led to an increase in mean pore size from 60.6 ± 25.4 µm to 70.5 ± 36.5 µm respectively. For PGS-M polyHIPEs, the mean pore size significantly decreased from 94.3 ± 70.0 µm to 51.8 ± 32.6 µm from 5% to 7% gelatin, respectively, and increased from 51.8 ± 32.6 µm to 59.0 ± 37.2 µm from 7% to 10%, respectively (Figure 1D).

With the inclusion of surfactant in the HIPE, increasing the concentration of the gelatin solution had no significant effect on the mean pore size of PCL-M or PGS-M polyHIPEs. Furthermore, there was no significant difference between surfactant-only and surfactant and gelatin polyHIPEs (Figures 1C, D).

For both PCL-M and PGS-M, 5% gelatin solution without additional surfactant resulted in the largest mean pore size (79.9 ± 42.9 µm and 94.3 ± 70.0 µm respectively), which are both significantly larger than conventional surfactant-only polyHIPEs (53.0 ± 18.7 µm and 27.7 ± 13.0 µm respectively) which are comparable to pore sizes of surfactant stabilised polyHIPEs commonly reported in literature (Kramer et al., 2021). Thus, all further analysis was completed on polyHIPE constructs fabricated using a 5% gelatin solution.

### 4.2 Porosity of polyHIPEs

All polyHIPEs had a reduced porosity compared to the theoretically predicted porosity of 82.1% and 88.4% for PCL-M and PGS-M respectively, with the largest decrease in porosity observed in surfactant-free, 5% gelatin polyHIPEs (Table 2).

**TABLE 2:** Porosity of PCL-M and PGS-M polyHIPEs, as measured via helium pycnometry. Porosity is expressed as a percentage of the total volume of the construct. The bottom row shows the theoretical porosity of all polyHIPEs fabricated (based on the volumes of liquids added to the initial emulsion) calculated using the ratio of internal phase volume to total emulsion volume.

Emulsion	PCL-M measured porosity (%)	PGS-M measured porosity (%)
G0S10	73	82
G5S10	74	78
G5S0	72	76
Theoretical (based on emulsion composition)	82	88

### 4.3 Mechanical characterisation of polyHIPEs

**FIGURE 2:**
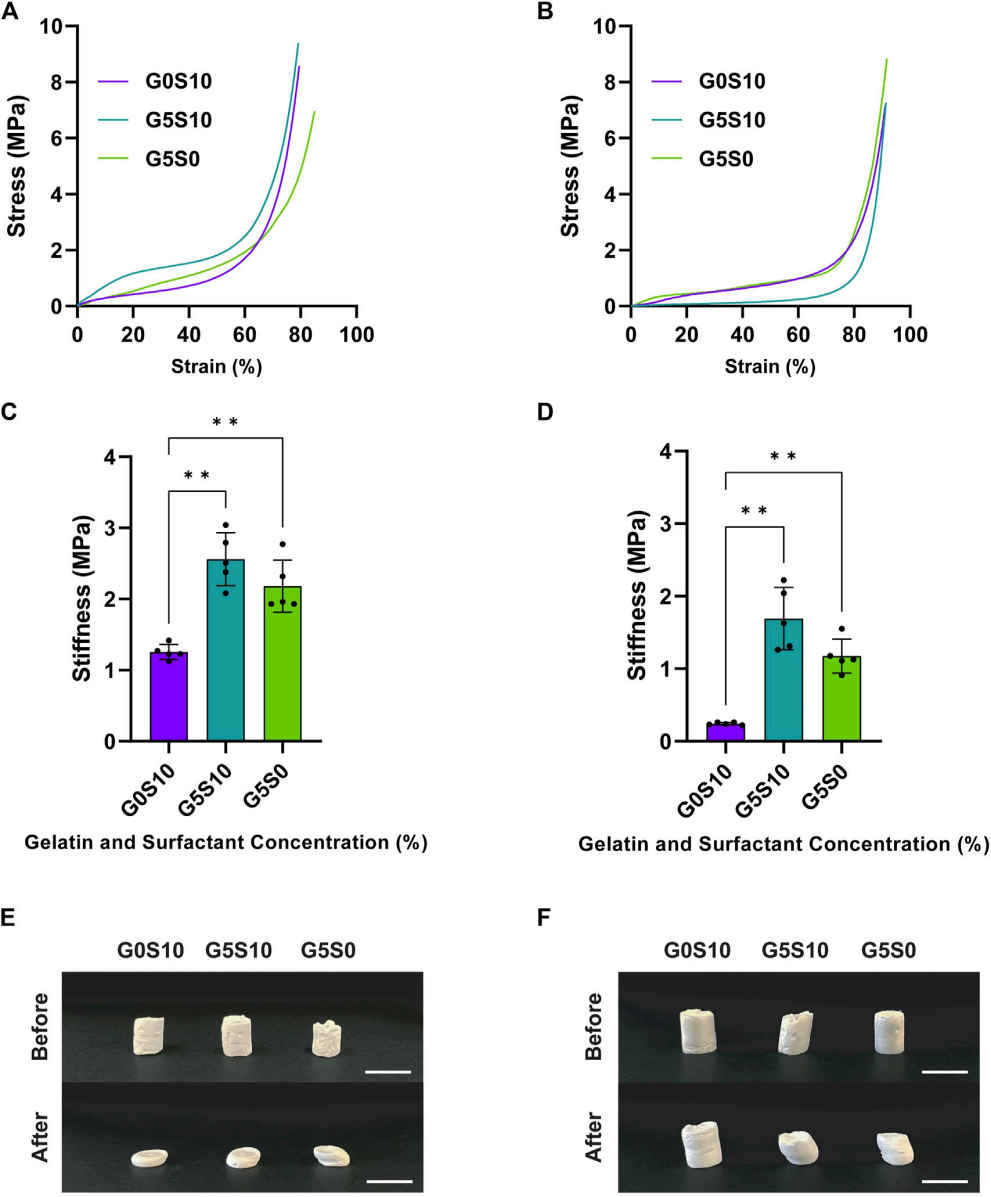
Representative stress-strain curves of **(A)** PCL-M and **(B)** PGS-M polyHIPEs following the removal of gelatin via acetic acid and freeze-drying with the corresponding mean stiffness of the **(C)** PCL-M and **(D)** PGS-M polyHIPEs (mean ± SD, *n* = 5, **p < 0.002). **(E)** PCL-M and **(F)** PGS-M polyHIPEs before and after mechanical compression under a 250 N load. PolyHIPEs from left to right are G0S10, G5S10, and G5S0 respectively (scale bar = 1 cm).

The inclusion of gelatin with or without surfactant in the formation of PCL-M and PGS-M polyHIPEs led to significantly higher stiffness than surfactant-only polyHIPEs (Figures 2A–D; Supplementary Table S1). There was no significant effect on stiffness with the addition of surfactant within gelatin constructs. Following deformation, all of the PCL-M polyHIPEs assessed exhibited little elastic recovery (Figure 2E). On the other hand, surfactant-only PGS-M polyHIPEs exhibited full elastic recovery following deformation, whereas PGS-M polyHIPEs fabricated with 5% gelatin solution exhibited reduced elastic recovery following deformation (Figure 2F).

### 4.4 Effect of washing on polyHIPE structures

**FIGURE 3:**
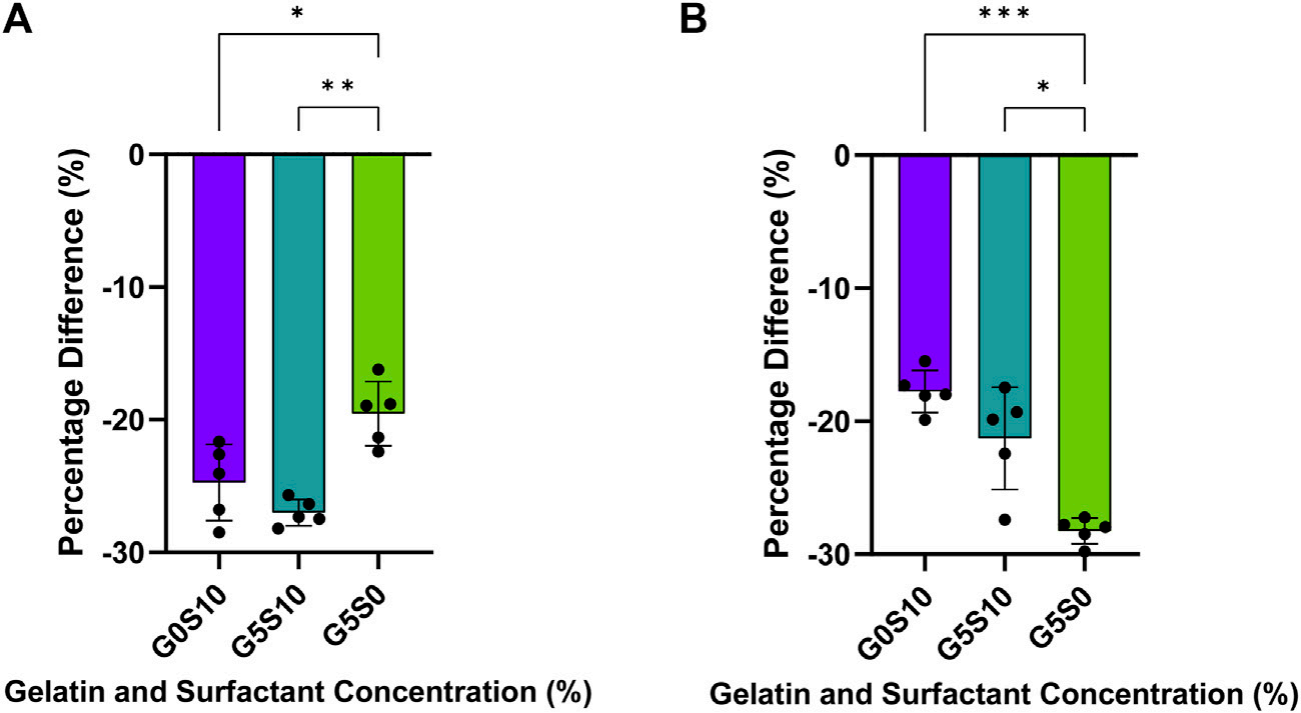
The percentage difference in the size of **(A)** PCL-M and **(B)** PGS-M polyHIPEs following the post-processing washing cycle (mean ± SD, n = 5, *p < 0.033, **p < 0.002, ***p < 0.001).

The polyHIPEs undergo several post-processing washing steps to remove excess solvent, surfactant and photoinitiator from within the structure. It was observed that through this post-processing cycle, the polyHIPEs significantly decreased in size, which was assumed to further cause a decrease in pore size (Figure 3). For both PCL-M and PGS-M surfactant-containing polyHIPEs the addition of gelatin did not significantly affect the degree of shrinkage following the washing process. However, for surfactant-free gelatin PCL-M, there was a significant reduction in shrinkage compared to surfactant-containing polyHIPEs (Figure 3A), whilst for surfactant-free gelatin PGS-M there was a significant increase in shrinkage compared to surfactant-containing polyHIPEs (Figure 3B).

### 4.5 Long-term stability of PCL-M and PGS-M emulsions

The amount of time an emulsion can remain stable without separating is affected by many factors such as surfactant type, concentration and emulsion composition, including that of the internal phase. PCL-M and PGS-M emulsions fabricated with 5% gelatin and stored at room temperature remained visibly stable for up to 56 days whereas surfactant-only emulsions stored at room temperature were stable for 24 h (Table 3). Visual stability was defined when there was no phase separation, flocculation or coalescence observed within the sealed storage vial which can usually be seen when instability occurs in emulsions (Tian et al., 2022) (Supplementary Figure S6). After storage at room temperature, the stable 5% gelatin emulsions became very viscous but were still able to be transferred into a mould for curing. However, after storage at 37°C for 24 h the 5% gelatin emulsions destabilised and phase separation was observed (data not shown).

**TABLE 3:** Long-term stability of G5S0 emulsions, fabricated and stored at room temperature prior to curing after 1 day, 7 days, 14 days, and 56 days. A conventional HIPE (G0S10) was used as a control. Emulsions were described as stable when no visible separation occurred and the emulsion could be transferred to a mould and photocrosslinked.

Polymer	Emulsion composition	Emulsion storage time			
		1 Day	7 Days	14 Days	56 Days
PCL-M	10% surfactant, 0% gelatin	Stable	Separation observed	Separation observed	Separation observed
	0% surfactant, 5% gelatin	Stable	Stable	Stable	Stable
PGS-M	10% surfactant, 0% gelatin	Stable	Separation observed	Separation observed	Separation observed
	0% surfactant, 5% gelatin	Stable	Stable	Stable	Stable

**FIGURE 4:**
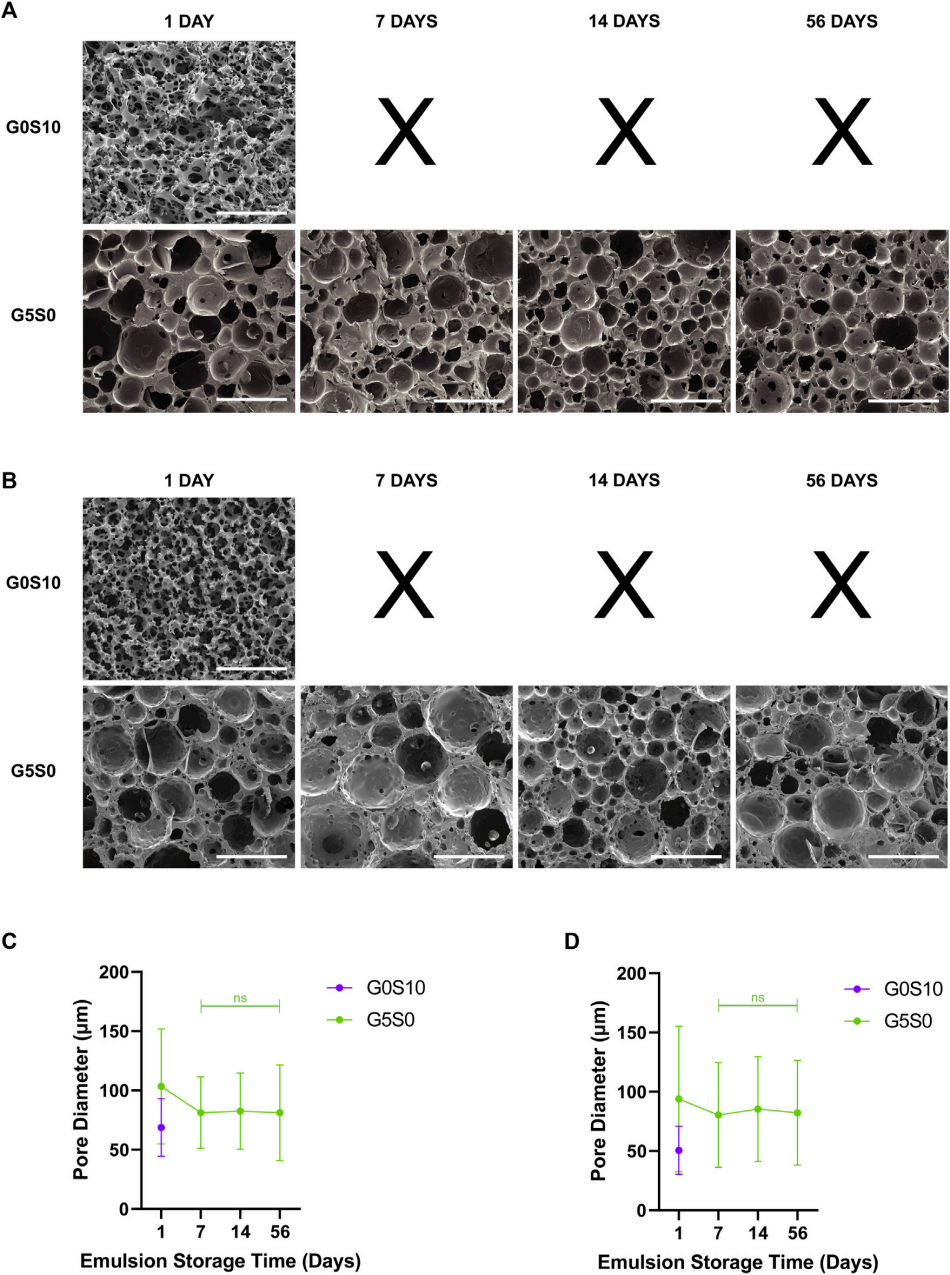
Scanning electron micrographs of **(A)** PCL-M and **(B)** PGS-M polymerised emulsions (polyHIPEs) fabricated with water and surfactant (G0S10) or with gelatin and no surfactant (G5S0), following storage of emulsions for 1 day, 7 days, 14 days, and 56 days, prior to curing (scale bar = 200 µm). Emulsions not containing gelatin were not fabricated and stored for more than 7 days following emulsion separation observed on day 7. The pore size of the resulting **(C)** PCL-M and **(D)** PGS-M surfactant-free gelatin polyHIPEs did not change significantly following storage of the emulsions from 7 days up to 56 days (mean ± SD, *n* = 60, ns = not significant).

The stable emulsions were cured and the micro-structures were further analysed via SEM. PCL-M and PGS-M emulsions cured after 24 h–56 days demonstrated typical porous polyHIPE structure in the resulting polyHIPE (Figures 4A, B). There was a decrease in mean pore size for both PCL-M and PGS-M polyHIPEs from day 1 to day 7 (Figures 4C, D). However, there was no significant difference between the pore sizes of polyHIPEs that were cured after being stored for 7, 14, and 56 days for both PCL-M and PGS-M surfactant-free gelatin polyHIPEs.

## 5 Discussion

This study investigated the effect of using a solution of gelatin as the internal phase in PCL-M and PGS-M polyHIPEs. Our findings indicate that gelatin can act as an emulsifier, and when used as the sole stabilising agent in the emulsion, it can generate polyHIPEs with large pore sizes (80–200 µm). This allows for the fabrication of surfactant-free polyHIPEs, whilst additionally providing a method of preloading the internal pores of polyHIPEs with gelatin. In addition, the use of gelatin impacts the mechanical properties of the polyHIPEs, resulting in a significant increase in stiffness. This effect was independent of the use of an additional surfactant (Hypermer B246) alongside gelatin. Furthermore, by adding gelatin to the internal phase, the emulsions can be stored for longer periods before curing compared to conventional emulsions. The resulting polyHIPEs fabricated from these stored emulsions retain their porous structure after curing, further enabling the potential applications of these gelatin-based emulsions.

The amphiphilic properties of gelatin have previously been harnessed to stabilise emulsions for the fabrication of polyHIPEs (Oh et al., 2015). Oh et al. (2015) used gelatin grafted onto poly (N-isopropyl-acrylamide) as the external phase of an o/w emulsion, with *p*-xylene as the internal oil phase, without additional surfactant. The grafted poly (N-isopropyl-acrylamide) side chains on the gelatin allowed the external phase of the emulsion to be solidified. These emulsions formed gelatinous scaffolds with macroporous structures. However, the study found that only the gelatin copolymer could stabilise the emulsion, not gelatin itself. It was also observed that the interfacial tension between grafted gelatin and *p*-xylene was lower than that between non-grafted gelatin and *p*-xylene. This may indicate that if the interfacial tension between the gelatin solution and the opposing phase is too high, gelatin may no longer act as an effective stabiliser.

Varying the concentration of gelatin within surfactant-free polyHIPEs allowed control over pore size. Using the lowest concentration of gelatin, 5%, conferred the largest mean pore size. When considering the pore size of polyHIPEs, it is worth noting that the constructs were imaged in dry conditions. PolyHIPEs can shrink following drying, in particular when they are formulated with porogenic solvents (Pierre et al., 2006; Murphy et al., 2017; Jackson et al., 2023). In this study, all constructs exhibited shrinkage following freeze drying (Supplementary Figure S5), so it should be considered that pore sizes in these dehydrated polyHIPEs may be smaller than if the polyHIPEs were hydrated.

As previously mentioned, the pore size of a polyHIPE can be controlled through emulsion stability, and as such, increasing surfactant concentration during polyHIPE fabrication increases stability and leads to a reduction in pore size. Following this principle, as gelatin behaves as a stabiliser (albeit a weak one), increasing the concentration of gelatin should lead to reduced polyHIPE pore size. This effect was observed in surfactant-free gelatin polyHIPEs, where the mean pore size from 5% to 7% gelatin decreased for PCL-M and PGS-M polyHIPEs. However, this trend was not maintained following an increase in gelatin concentration from 7% to 10% gelatin, where the pore size instead increased slightly. As increasing gelatin concentration increases the viscosity of the gelatin solution (Sancakli et al., 2021), the increase in pore size between 7% and 10% gelatin may be due to increased viscosity of the internal phase. Increased internal phase viscosity compared to external phase viscosity has been shown to inhibit efficient mixing (Busby et al., 2001; Kataruka and Hutchens, 2019). Reduced mixing efficiency reduces droplet transport and distribution throughout the emulsion, preventing droplet breakup from collision with the stirrer (Sajjadi, 2006; Ashrafizadeh and Kamran, 2010). In addition, with reduced mixing efficiency, droplets undergo reduced shear forces (Moglia et al., 2014), which in turn may reduce droplet dispersal, leading to increased droplet size and subsequently larger pore sizes. The effect of reduced mixing efficiency and subsequent droplet breakup may also be exacerbated by the low mixing speed used within this study of 250 rpm (Moglia et al., 2011; Jackson et al., 2023). Thus, increasing gelatin concentration and its stabilising ability leads to a reduction in droplet size, however, when a critical viscosity of gelatin solution is reached, viscosity has a greater influence on polyHIPE pore size than increasing gelatin concentration.

Whilst pore size is a key factor in construct design, another important parameter is porosity, which is the percentage of void space within the material. Here we investigated the porosity of gelatin polyHIPEs using helium pycnometry. It was observed that the measured porosity was less than the theoretical porosity. All the emulsions had the same volume of internal phase added, therefore any decrease in measured porosity of the resulting polyHIPEs from the theoretical value is not likely due to a reduction in internal pores, but instead a reduction in interconnectivity. This is further supported by the SEM characterisation (Figures 1A, B), where more interconnects are visible in the surfactant-stabilised polyHIPEs compared to the gelatin-stabilised polyHIPEs. Any closed pores within the polyHIPE will reduce the measured porosity. The relationship between interconnectivity and porosity in polyHIPEs has been previously reported (O’Brien, 2011; Aldemir Dikici and Claeyssens, 2020). For example, Owen et al. (2016) observed a linear relationship between porosity and degree of openness (interconnectivity). While the incorporation of gelatin in the internal phase during polyHIPE fabrication slightly reduced porosity in PCL-M and PGS-M polyHIPEs, the materials remained highly porous (>70%).

PolyHIPEs are commonly subjected to several post-processing steps to remove unreacted monomer, excess solvent, surfactant and photoinitiator from within the structure. These steps are especially important for tissue engineering applications to prevent the leaching of cytotoxic chemicals. Washing of polyHIPEs can cause shrinkage or swelling, depending on the material composition as well as the medium used for washing (Aldemir Dikici and Claeyssens, 2020). For design and manufacturing purposes, it is important to know the degree of swelling or shrinkage of a certain polyHIPE, so that size of moulds can be scaled to account for the change in construct size.

A degree of shrinkage was observed across all polyHIPEs between the fabrication of the polyHIPE and after the post-processing steps (Figure 3). These steps consisted of an acetic acid wash to remove gelatin, followed by washing and rehydration of the polyHIPE through a series of ethanol dilutions and water. It has been reported that polyHIPEs can reduce in size due to pore collapse following the removal of solvent (Woodward et al., 2017). However, the SEM images of the washed samples did not reveal that the gelatin polyHIPEs exhibited structural collapse following washing, indicating that their shrinkage may instead be due to the elution of the solvent during water washes. This principle has been previously utilised by Dikici et al. (2020), where the shrinkage of polyHIPEs was used to aid in the assembly of a bilayer tube. Many reports have focused on the shrinkage of polyHIPEs due to drying (Hobiger et al., 2021), although there has been less focus on shrinkage during washing. However, shrinkage and swelling have been noted for gels and colloids during washing (Nussinovitch and Peleg, 1990).

Mechanical strength is an important characteristic, as it determines the ability of a material to withstand external forces and loads without failure. Overall, the inclusion of gelatin as the internal phase during the fabrication of polyHIPEs increased the compressive mechanical strength compared to conventional surfactant-stabilised polyHIPEs (G0S10) (Figure 2). It is well established within the literature that for conventional polyHIPEs, larger pore sizes confer greater mechanical strength than smaller pore sizes, usually due to the increased thickness of the walls between pores (Jiang et al., 2007; Lin-Gibson et al., 2007; Huš and Krajnc, 2014; Aldemir Dikici et al., 2019; Kovačič et al., 2019). However, concerning the use of co-emulsifiers, Wu et al. (2010) report that the inclusion of co-emulsifying silica nanoparticles alongside a surfactant increased mechanical strength compared to conventional polyHIPEs, despite a reduction in pore size. Therefore, we might expect that the use of both surfactant (hypermer) and gelatin as co-emulsifiers would lead to a synergistic emulsifying effect, resulting in smaller pore sizes and increased mechanical strength. However, we observed that the inclusion of gelatin and surfactant did not change mean pore size compared to conventional polyHIPEs (Figures 1C, D), despite an increase in stiffness (Figure 2). Furthermore, G5S10 and G5S0 polyHIPEs had similar mechanical properties, whilst having different pore sizes. This may indicate that the inclusion of gelatin during fabrication had a larger overarching effect on mechanical properties than the change in pore size.

In the literature, several methods have been established to alter the mechanical properties of polyHIPEs, which can be divided into three categories; modifications to the emulsion parameters, modification of the polymerisation and post-processing modifications. During emulsification, internal phase volume (Aldemir Dikici and Claeyssens, 2020), surfactant type and concentration (Aldemir Dikici et al., 2022b), mixing speed (Jackson et al., 2023), diluting solvent type and ratio (Aldemir Dikici et al., 2019), and the inclusion of reinforcing agents or particles in the external phase (Wu et al., 2010) can all alter the mechanical properties of the resulting polyHIPE. However, these mechanisms also alter the pore size of the polyHIPE, which may limit their benefit for some applications. The polymerisation process can also be modified. For example, Luo et al. (2012) used reversible addition-fragmentation chain transfer polymerisation to increase the mechanical properties of styrene and divinylbenzene polyHIPEs 3-fold compared to conventional radical polymerisation techniques. However, this resulted in varying pore morphology. Finally, post-processing steps can be performed on polyHIPEs to increase their mechanical properties. For example, electroless nickel plating has been used to coat the surface of polyHIPEs, conferring a >4-fold increase in stiffness (Sengokmen-Ozsoz et al., 2023). However, this leads to reduced surface porosity and increases the complexity of fabrication with additional processing steps. In comparison to the above-mentioned techniques, using the methodology presented in this study, combining surfactant and gelatin, we provide a simple technique to increase mechanical strength whilst maintaining the pore size. This method does not require alteration to the composition of the external phase, or the polymerisation reaction or necessitate additional post-processing steps. Emulsions are typically metastable, stabilised by surfactants or particles to lower the interfacial tension, however, they eventually undergo phase separation. The lifetime for which an emulsion can remain stable (referred to in this study as long-term stability) depends on the formulation of the emulsion. The final “breaking” of the emulsion occurs through various mechanisms which have been widely studied such as creaming, flocculation, Ostwald ripening and coalescence (Ostwald, 1901; Friberg et al., 1976; Pays et al., 2010; McClements and Jafari, 2018).

The ability to store uncured emulsions for a long time may be beneficial for use as an inherently porous photocurable resin, allowing an emulsion to be fabricated, transported, stored and later cured into a desired shape by the end user. However, the long-term stability of polymeric emulsions can be short-lived and thus may be insufficient for this purpose (Pays et al., 2010). When 5% gelatin solution was used as the internal phase, emulsions were stable for 56 days, whereas a conventional emulsion without gelatin was unstable by day 7. Given that gelatin solution transitions to a gel state at lower temperatures, after fabrication of the emulsions at 37°C, gelatin solidifies, and therefore the droplets of the internal phase are no longer in a liquid state. Therefore, it is no longer as energetically favourable for the gelatin droplets to coalesce, and the solid droplets remain suspended and stable in the viscous emulsion, unlike a standard emulsion where both phases remain liquid. Oh et al. (2015) observed a similar effect in emulsions fabricated using a gelatin copolymer as the external phase. Following cooling of the emulsions to 4°C, the viscosity of the emulsions increased and they did not flow any more, remaining kinetically stable; this was attributed to the gelation of gelatin.

The internal phase of w/o emulsions is most commonly composed of water (Cameron, 2005), and, following this study, it may be the case that by simply substituting this water for a solution of gelatin and allowing it to cool, the storage time of these emulsions before curing could be increased. This enhanced long-term stability could also provide further opportunities for polyHIPE fabrication, such as the solidification of polyHIPEs without the need for polymerisation. Polymerisation-free polyHIPE solidification is a process based on solvent evaporation, and is usually limited by the long solidification process, which can take 24–48 h (Samanta et al., 2016; Yang et al., 2017), and thus requires stability to be maintained over this period. However, with gelatin as the internal phase, stability could be maintained, even if the initial stability of the emulsion before cooling was weak.

In this study, gelatin was removed from the polyHIPEs before characterisation. We aimed to investigate how using gelatin during polyHIPE fabrication impacted the resulting structure of the polymer, rather than study the composite polymer-gelatin structure. However, for some applications, it may be beneficial to retain the gelatin within the structure, for example, if used as a bioprinting ink with the inclusion of living cells within the gelatin solution. Retaining the gelatin within polyHIPEs may have environmental applications. Gelatin has been utilised to treat wastewater used for irrigation, to prevent the accumulation of pollutant heavy metal ions in agricultural soil (Chen et al., 2014; Li et al., 2016). In this instance, amino groups in gelatin bind to the metal ions via chelation. Using gelatin as the internal phase of polyHIPEs may be a simple fabrication method to create “sponges” that can be used within wastewater filtration systems to absorb pollutants, preventing soil contamination. Furthermore, gelatin has been shown to provide a source of nitrogen, acting as a biostimulant seed treatment, improving plant performance (Wilson et al., 2018). Incorporation of gelatin within a polymer matrix such as a polyHIPE could provide ease of handling and structural integrity, as well as protect the gelatin from degradation via external factors such as UV light. Over time, the polymer would degrade, slowly releasing the gelatin in a manner which could be controlled by tailoring the degradation rate of the polymer. Whilst there are more cost-effective traditional fertilisers available, there are other beneficial factors of a polyHIPE delivery system, including, controlled release and reduction in fertiliser run-off.

Gelatin is commonly used as a coating for tissue engineering scaffolds as it contains the arginine–glycine–aspartic acid (RGD) sequence, a key molecule for the formation of interactions between integrins on the cell surface and the surrounding extracellular matrix (Kim et al., 2017). In addition, most polymers used in conventional w/o polyHIPEs are hydrophobic (Cameron, 2005), which can reduce cell adhesion on the material. These hydrophobic polymers commonly require surface modification or coating to improve their hydrophilicity (Qin et al., 2022). Gelatin is a hydrophilic molecule, and if used as the internal phase for a polyHIPE made from a hydrophobic polymer, it could potentially improve initial cell attachment, and provide a simple method for internally pre-coating the pores of a scaffold for tissue engineering purposes. In addition, the gelatin could be loaded to release bioactive factors to promote tissue-specific cellular responses such as cell proliferation and differentiation (Santoro et al., 2014).

Similarly, gelatin-loaded polyHIPEs could be used to create sustained-release drug delivery systems where the gelatin acts as a drug carrier (Milano et al., 2023) For example, Toyama et al. (2012) have performed a clinical trial using cisplatin-loaded gelatin microspheres to treat liver carcinoma, with a 100% success rate. By using emulsion templating to incorporate the gelatin into the pores of a polyHIPE, the material could be pre-loaded with gelatin. The external polymer could provide mechanical strength and stability to the drug delivery system, and the internal gelatin could provide the functional aspect of drug delivery. However, the safety and efficacy of gelatin drug delivery systems needs to be established.

## 6 Conclusion

In summary, in this study, we demonstrated that gelatin solution can be used as the internal phase of w/o HIPEs. The gelatin within the internal phase has the ability to stabilise the emulsion, and thus, PCL-M and PGS-M emulsions can be fabricated without the need for an additional surfactant. The resultant polyHIPEs had significantly increased pore size, which could be altered by changing the concentration of the gelatin solution. Furthermore, the utilisation of gelatin within the internal phase increased the mechanical properties of the polyHIPEs, while maintaining a high porosity of 72% and 76% for PCL-M and PGS-M respectively. Despite gelatin being a weak stabilising agent, gelatin-containing emulsions displayed improved long-term stability at room temperature compared to conventional emulsions, which we attribute to an increase in the viscosity of gelatin as a result of the gelation of gelatin at lower temperatures. These findings suggest that gelatin has great potential to be used as a stabiliser for the production of surfactant-free polyHIPEs with tuneable macroporous structures. Surfactant-free gelatin polyHIPEs may hold promise in numerous applications, and highlight the potential use of amphiphilic natural polymers as an alternative stabiliser for the generation of polyHIPE constructs with large pore structures.

## Data Availability

The raw data supporting the conclusion of this article will be made available by the authors, without undue reservation.
