# Supplementary material for: Surfactant-free gelatin-stabilised biodegradable polymerised high internal phase emulsions with macroporous structures
Source: Front Chem. 2023 Aug 23;11:1236944. doi: 10.3389/fchem.2023.1236944 (PMC10481965; doi:10.3389/fchem.2023.1236944)
Supplement: Supplementary file 1 [file DataSheet1.docx]

***Supplementary Material***

Surfactant-Free Gelatin-Stabilised Biodegradable Polymerised High Internal Phase Emulsions (PolyHIPEs) with Macroporous Structures

**Rachel Furmidge^1,2†^, Caitlin E. Jackson^1,2†^, María Fernanda Velázquez de la Paz^1,2^, Victoria L. Workman^1,2^, Nicola H. Green^1,2^, Gwendolen C. Reilly^1,2^, Vanessa Hearnden^1,2^, Frederik Claeyssens^1,2^***

*** Correspondence:** Corresponding Author: [f.claeyssens@sheffield.ac.uk](mailto:f.claeyssens@sheffield.ac.uk)

# Supplementary Methods, Figures and Tables

# Characterisation of PCL-M and PGS-M using gel permeation chromatography (GPC)

Gel permeation chromatography (GPC) was used to determine the molecular weights of PCL-M and PGS-M. The samples were dissolved in tetrahydrofuran (0.10 mg/mL) and injected at a flow rate of 1 mL/min into a Viscotek GPCmax (VE2001), with a differential refractive index detector (Waters 410). Toluene was added as a reference and samples were analysed in a 650 mm PLgel 3 μm mixed E column at 40°C. Chromatogram peaks were analysed to determine the molecular weight average (*M*w) and the polydispersity index (PD).

# Characterisation of PCL-M and PGS-M by proton nuclear magnetic resonance (NMR) spectroscopy

The methacrylation of PCL-M and PGS-M was determined using proton (1H) nuclear magnetic resonance (NMR) spectroscopy. The spectrometer (Burker AVIIIHD 400 NMR spectrometer) frequency was set at 400MHz and data was recorded using a 30° pulse for excitation, 64k acquisition points over a spectral width of 20.5 ppm, 64 transients and a relaxation delay of 2 s. Samples were dissolved in 1 mL of deuterated chloroform at 1% w/v. Chemical shifts were referenced to the deuterated chlorofrom at 7.27 ppm. Spectra were analysed using MestReNova software (Version 11.0.4-18998, Mestrelab Research). The degree of methacrylation of PCL-M was determined by comparing the integrals of the methacrylate group peaks (5.5 and 6 ppm) to the peaks of the hydroxyl groups (3.6 ppm). The degree of methacrylation for PGS-M was determined by comparing the integrals of the methacrylate group peaks (1.9, 5.6 and 6.2 ppm) to the sebacic acid peak (1.3 ppm).


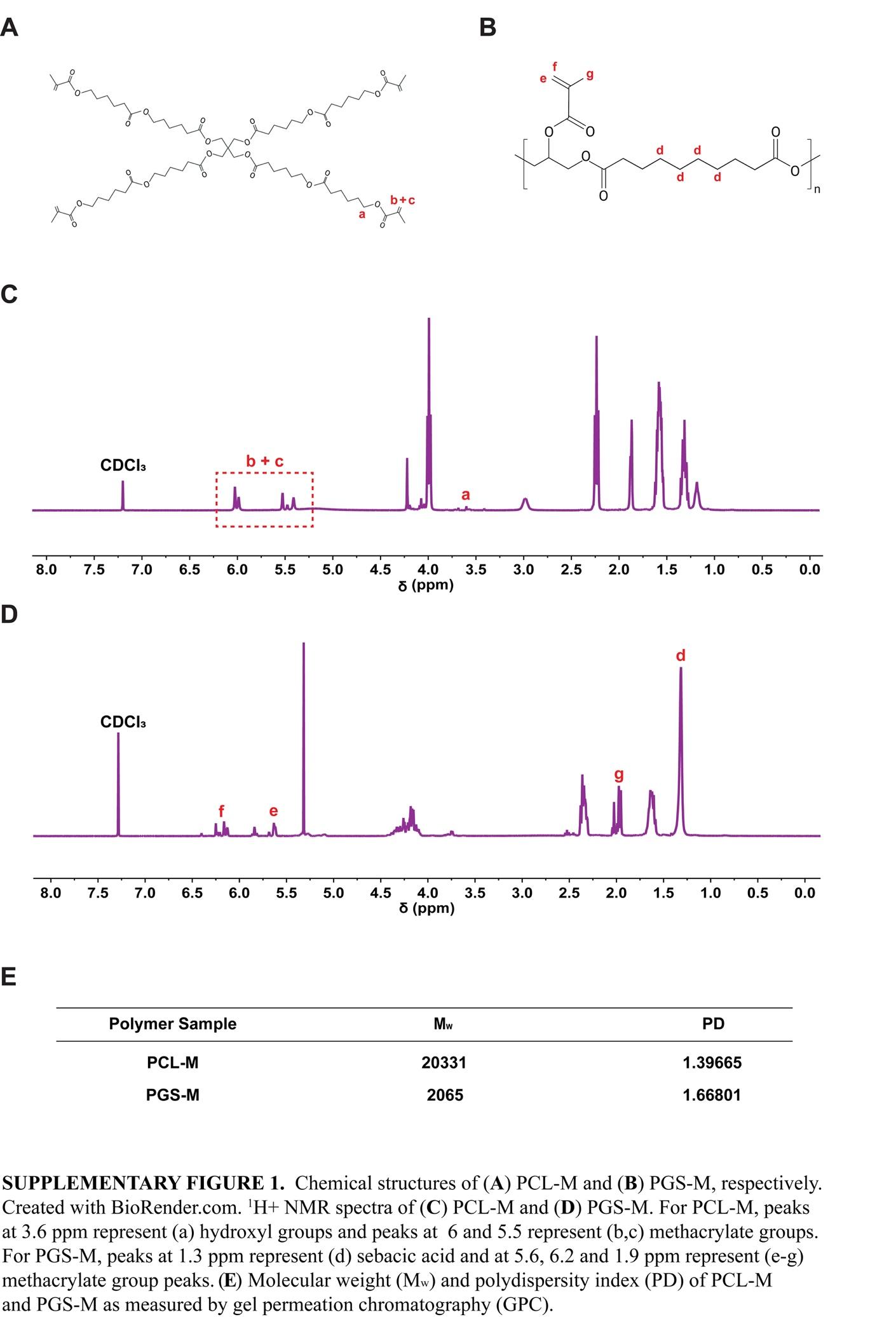


**SUPPLEMENTARY FIGURE 1.** Chemical structures of (A) PCL-M and (B) PGS-M, respectively. Created with BioRender.com. 1H+ NMR spectra of (C) PCL-M and (D) PGS-M. For PCL-M, peaks at 3.6 ppm represent (a) hydroxyl groups and peaks at 6 and 5.5 represent (b,c) methacrylate groups. For PGS-M, peaks at 1.3 ppm represent (d) sebacic acid and at 5.6, 6.2 and 1.9 ppm represent (e-g) methacrylate group peaks. (E) Molecular weight (Mw) and polydispersity index (PD) of PCL-M and PGS-M as measured by gel permeation chromatography (GPC).

**
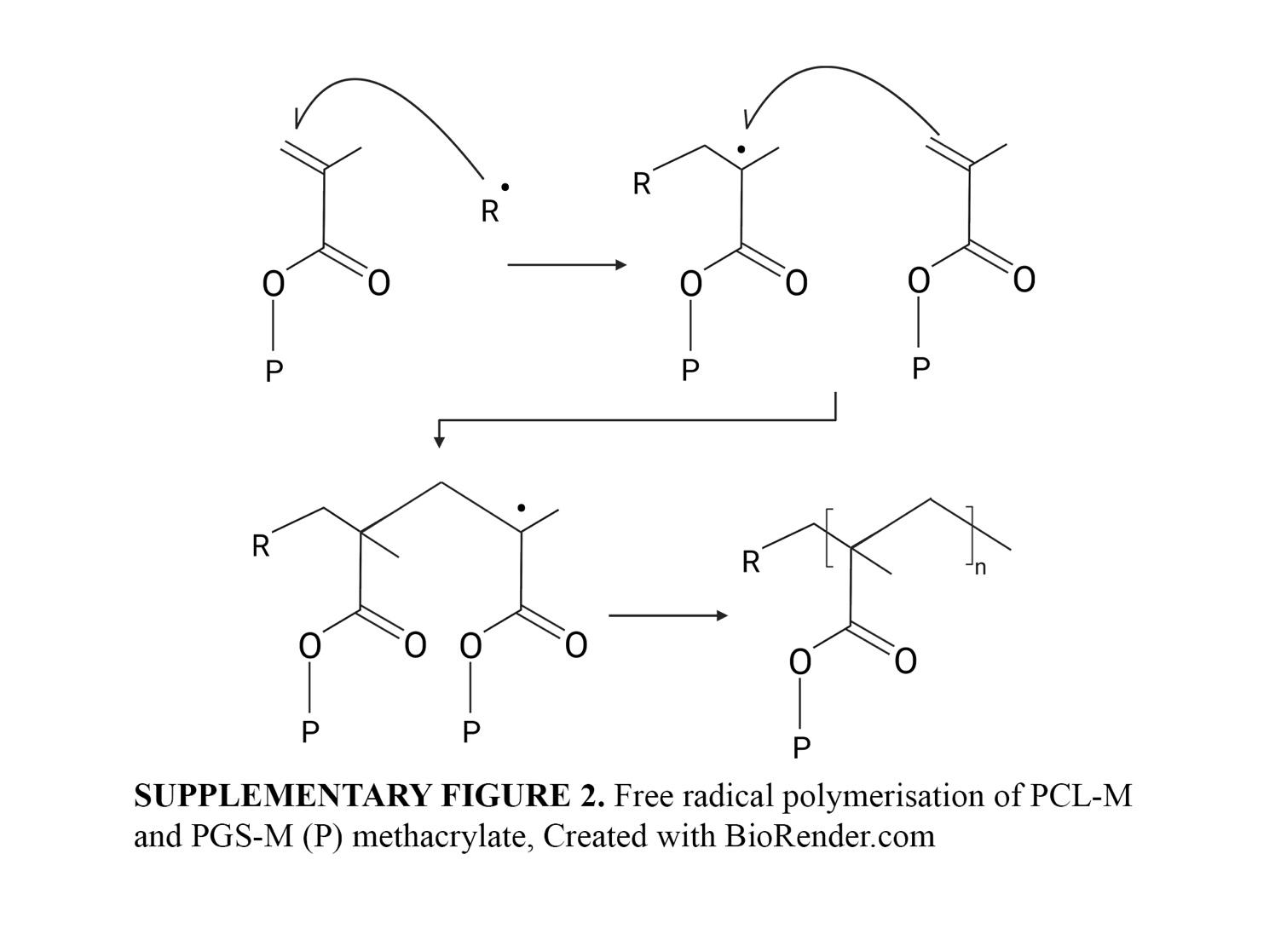
**

**SUPPLEMENTARY FIGURE 2.** Free radical polymerisation of PCL-M and PGS-M (P) methacrylate, Created with BioRender.com


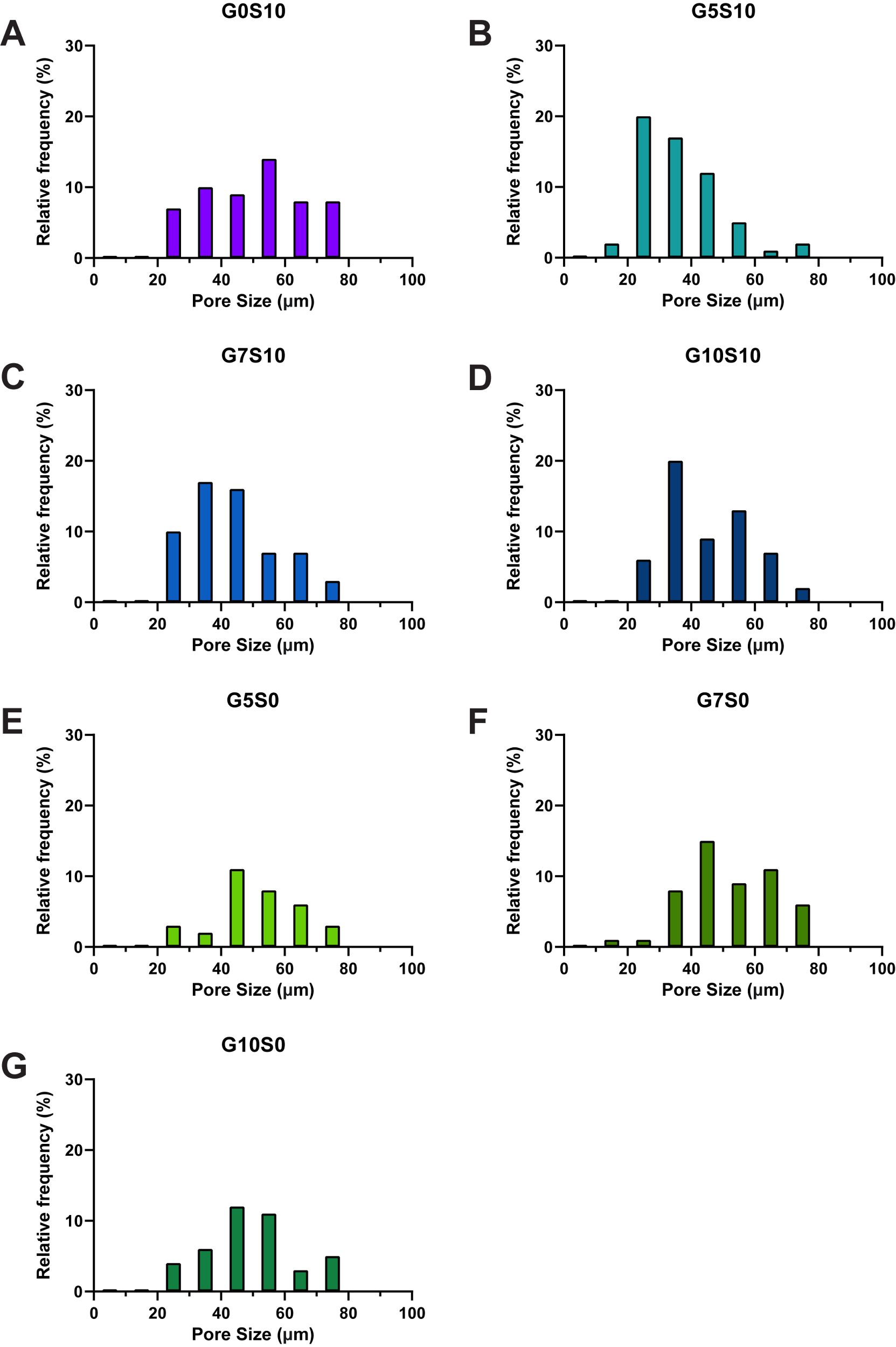


**SUPPLEMENTARY FIGURE 3.** Relative frequency and distribution of pore sizes in PCL-M polyHIPEs fabricated using different concentrations of gelatin ± surfactant.

**
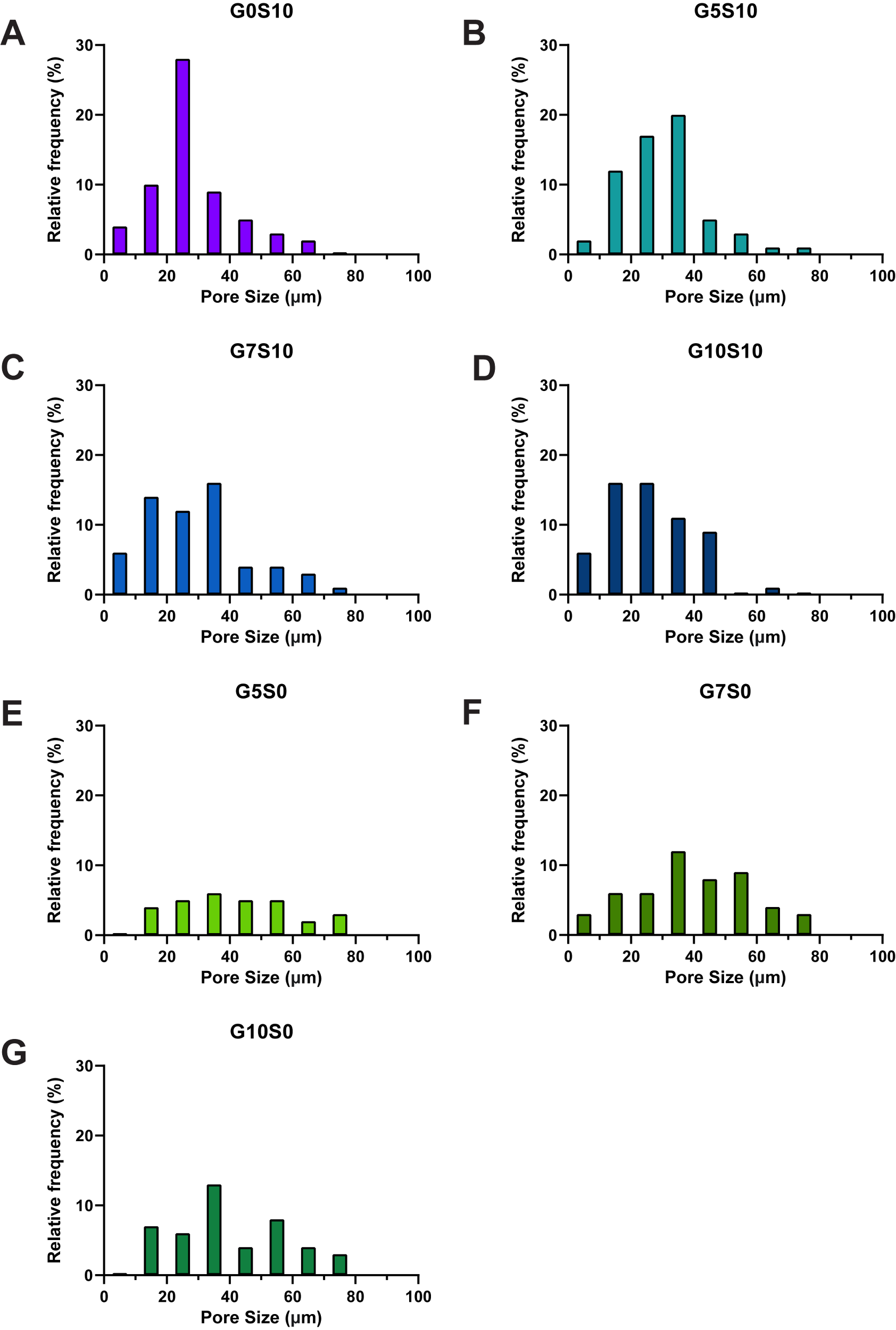
**

**SUPPLEMENTARY FIGURE 4.** Relative frequency and distribution of pore sizes in PGS-M polyHIPEs fabricated using different concentrations of gelatin ± surfactant.

**SUPPLEMENTARY TABLE 1.** Mean (± SD) stiffness of PCL-M and PGS-M polyHIPEs following the removal of gelatin via acetic acid and freeze drying (*n* = 5).

| **Emulsion** | **Mean Stiffness (MPa)** | |
| --- | --- | --- |
|  | **PCL** | **PGS** |
| G0S10 | 1.26 ± 0.11 | 0.24 ± 0.018 |
| G5S10 | 2.56 ± 0.37 | 1.69 ± 0.43 |
| G5S0 | 2.18 ± 0.37 | 1.18 ± 0.23 |

**SUPPLEMENTARY FIGURE 5.** The percentage difference in the size of **(A)** PCL-M and **(B)** PGS-M polyHIPEs from a wet to dry state following freeze drying (mean ± SD, *n = 5, * p <* 0.033, *** *p <* 0.001).
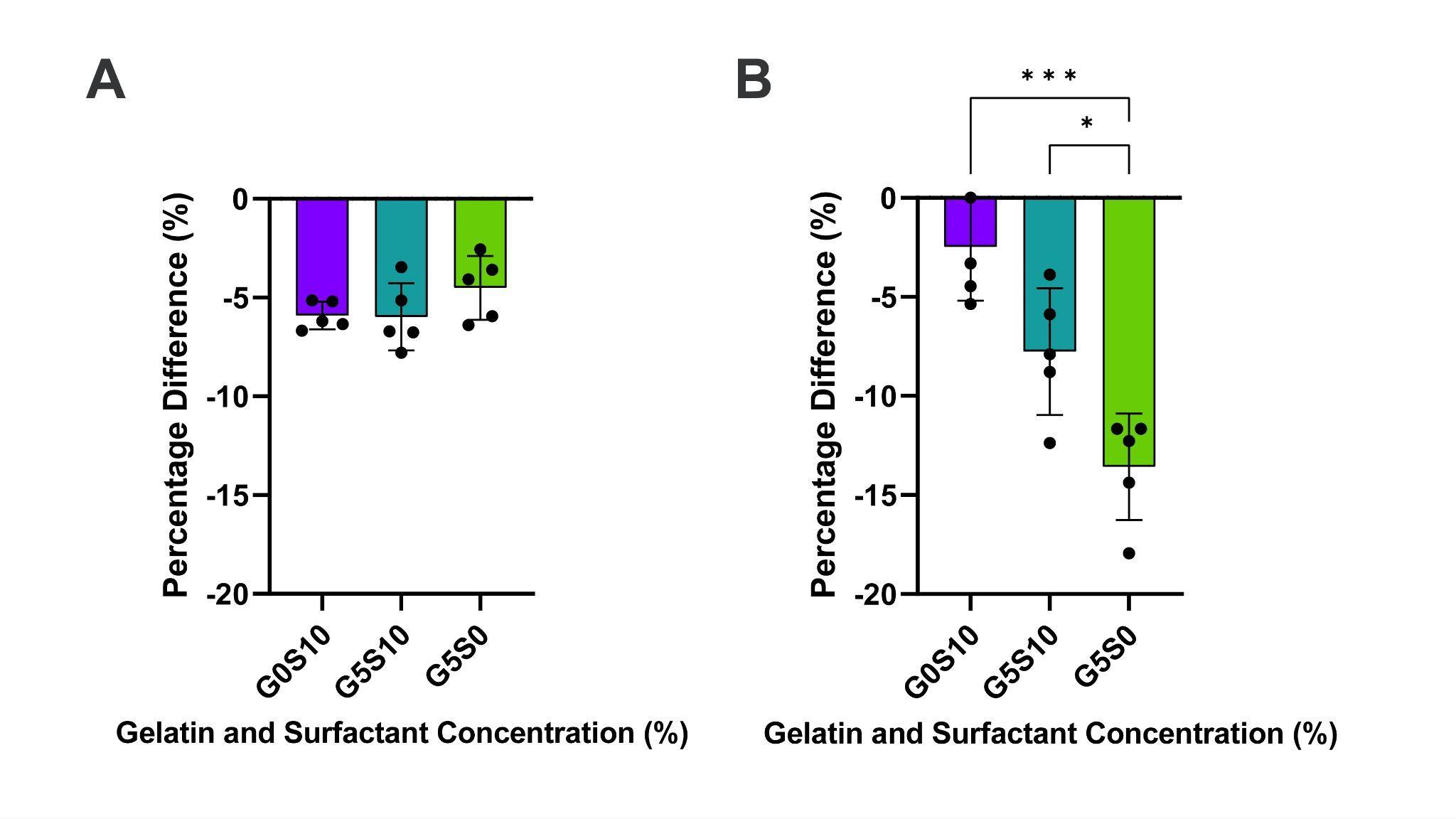


**
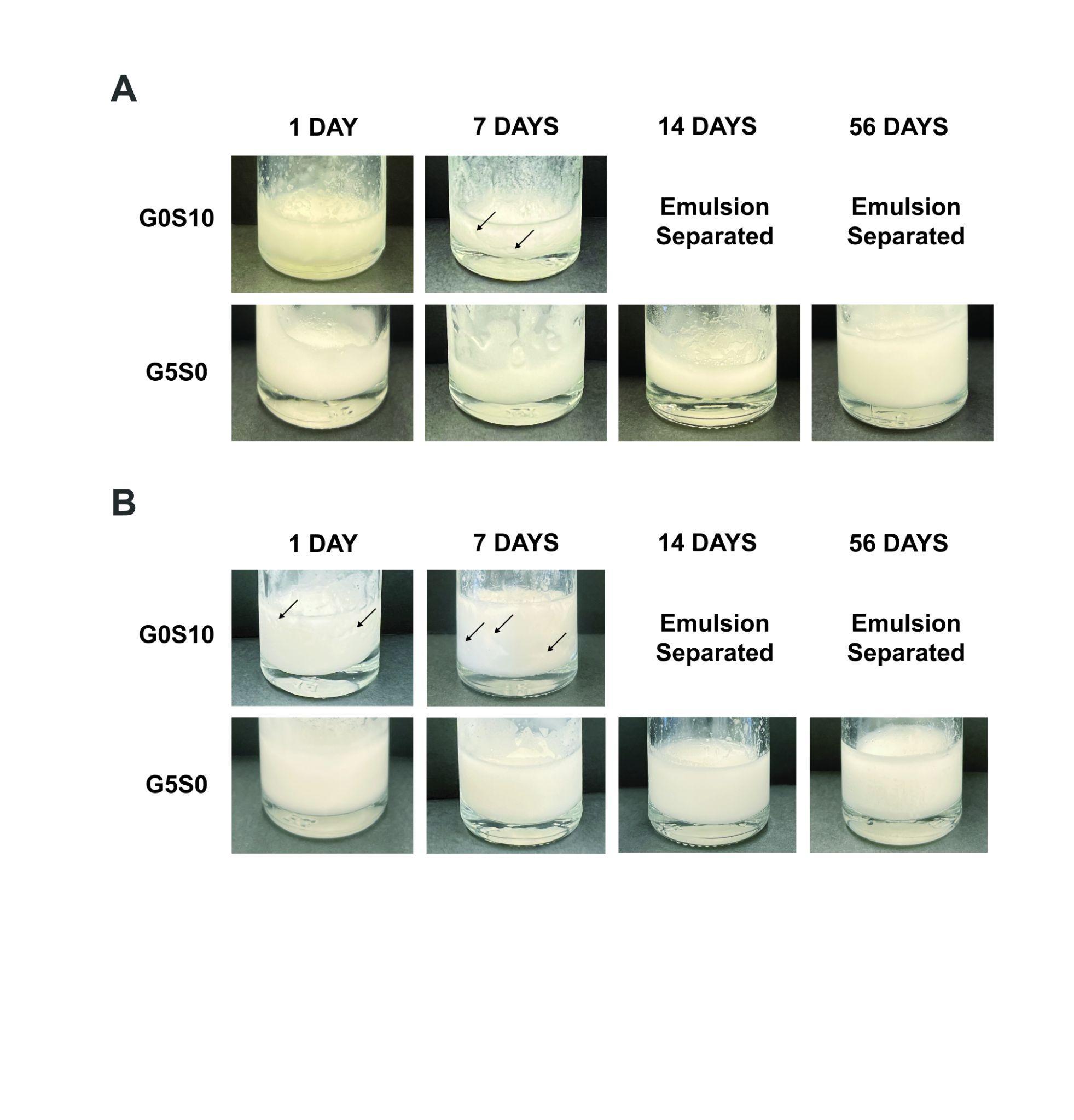
**

**SUPPLEMENTARY FIGURE 6.** **(A)**  PCL-M and **(B)** PGS-M emulsions fabricated with water and surfactant (G0S10) or with gelatin and no surfactant (G5S0), following storage for 1 day, 7 days, 14 days and 56 days. Arrows indicate areas of visible phase separation. Emulsions not containing gelatin were not fabricated and stored for more than 7 days following emulsion separation observed at day 7.
